# Supplementary material for: Optical vagus nerve modulation of heart and respiration via heart-injected retrograde AAV
Source: Sci Rep. 2021 Feb 11;11:3664. doi: 10.1038/s41598-021-83280-3 (PMC7878800; doi:10.1038/s41598-021-83280-3)
Supplement: Supplementary file 1 — Supplementary Information. [file 41598_2021_83280_MOESM1_ESM.docx]

**Optical Vagus Nerve Modulation of Heart and Respiration via Heart-Injected Retrograde AAV**

Arjun K. Fontaine^1,3*^, Gregory L. Futia^1,*^, Pradeep S. Rajendran^4,5^, Samuel Littich^1,3^, Naoko Mizoguchi^2,7^, Kalyanam Shivkumar^4,5^, Jeffrey L. Ardell^4,5^, Diego Restrepo^2,†^, John H. Caldwell^2,†^, Emily A. Gibson^1,†^, Richard F. *ff* Weir^1,3,6,†^

**Supplementary Figures**


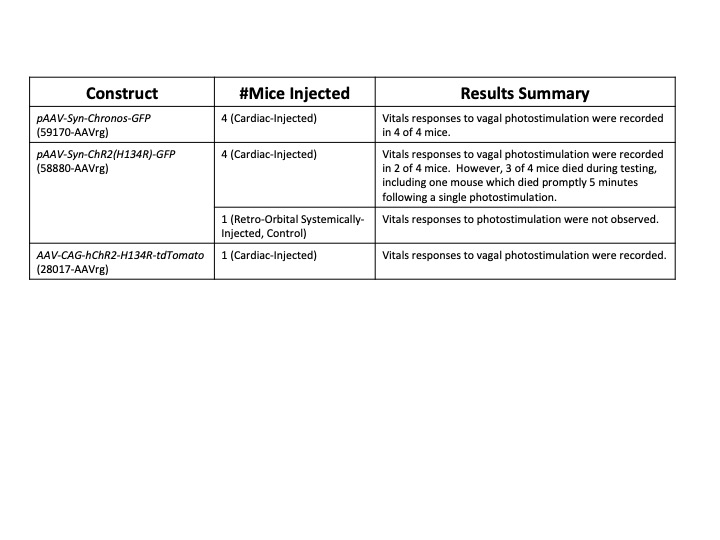


Supplementary Figure 1: Summary table of mice injected with retro-AAV and study results.


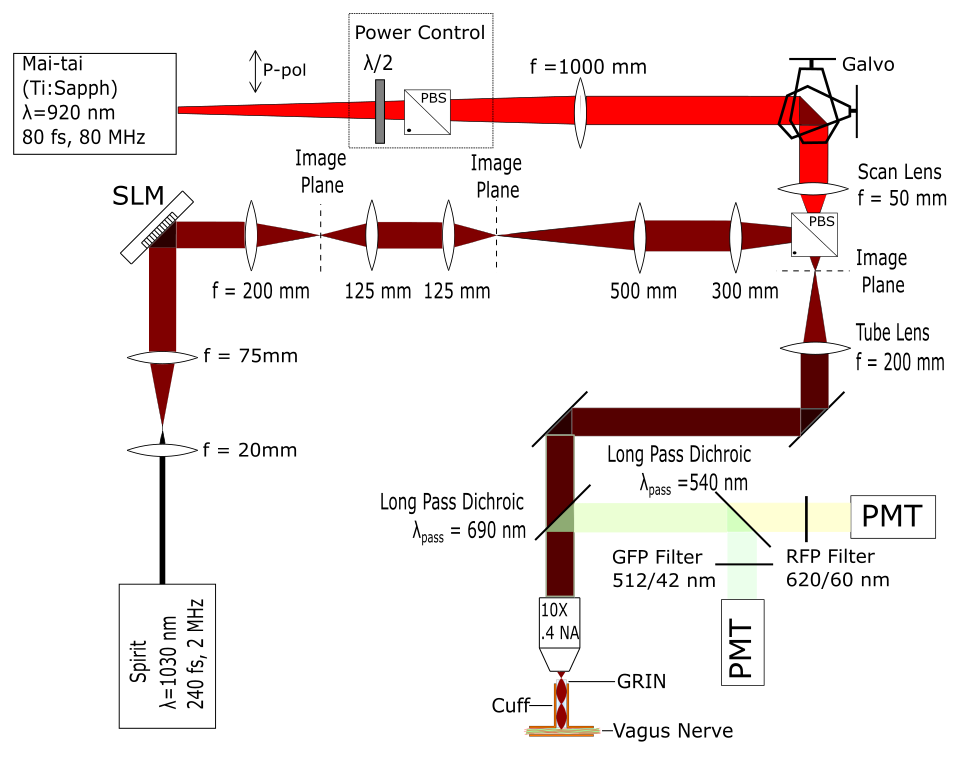


Supplementary Figure 2: Diagram of two-photon imaging and holographic photostimulation setup showing the beam path for imaging and a separate path for photostimulation using a spatial light modulator for shaping the intensity pattern at the focus. The beams are combined after the scan lens using a polarizing beam splitter. The GRIN-cuff is positioned under the air objective to focus into the cervical vagus nerve.

Supplementary Figure 3: Transverse (xy) and axial (xz, yz) profiling of holographic two-photon excitation at the focus of the GRIN relay lens within the vagus nerve. (A) spatial profile for two 10 μm spots (B-D) excitation profiles used for the *in vivo* studies reported in Fig. 6. The axial FWHM were calculated from the z stacks to be (A) 44 microns, (B) 105 microns (left region) 140 microns (right region), (C) 179 microns, and (D) 344 microns. Images were acquired by translating the two-photon holograms being formed at the object plane of the GRIN lens through a thin fluorescent slide with emission imaged with stationary inspection microscope onto a camera.


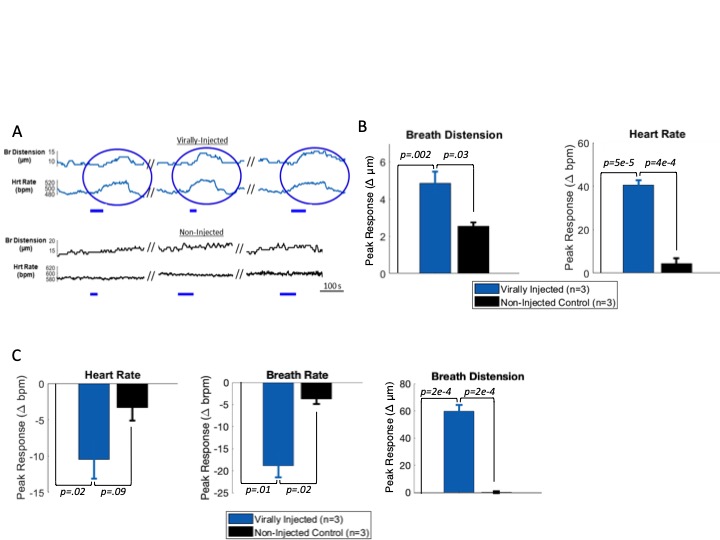


Supplementary Figure 4: (A) Vitals response to 1-photon stimulation shown in Figure 4B of manuscript. Signal quantification for vitals responses to (B) 1-photon stimulation of the cervical vagus nerve shown in Figure 4B, and (C) 2-photon holographic photostimulation shown in Figure5A&B. Amplitudes are calculated as the peak measurement during the post-stimulus window minus the mean pre-stimulus baseline. (Minor linear trends in control traces were not de-trended, contributing to the non-zero peak response).


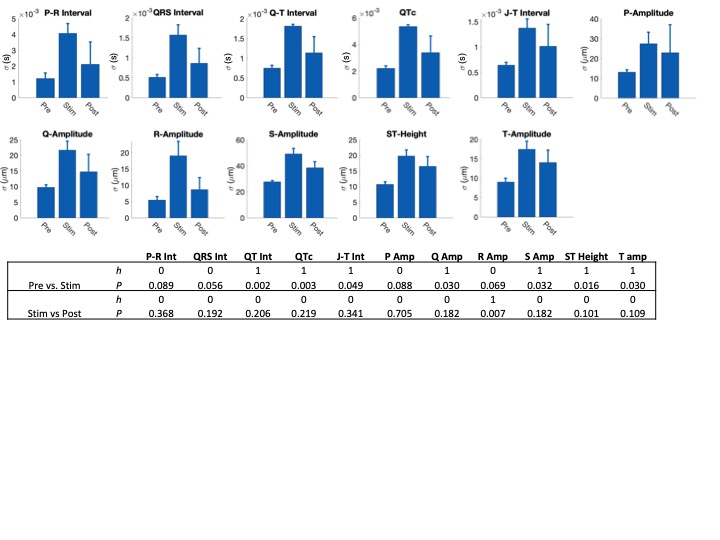


Supplementary Figure 5: Standard deviation of ECG parameters for n=3 ‘virally-injected’ signals presented in Figure 4D. Quantification of ECG parameter standard deviation is shown during the pre-stimulus state (Pre), the period in which heart rate is perturbed > 5% from baseline (Stim), and the period following the heart rate perturbation (Post), along with statistical test data (paired t-test) for difference in these standard deviations. Standard deviation is significantly modulated for many of the ECG parameters measured, particularly between the pre-stimulus baseline and stimulus period. In the ‘non-injected’ control case, for n=3 signals presented in Figure 4D there is no significant difference in ECG parameter standard deviation across pre, stim, and post periods (data not shown).
